# Immune Derangements in Patients with Myelofibrosis: The Role of Treg, Th17, and sIL2Rα

**DOI:** 10.1371/journal.pone.0116723

**Published:** 2015-03-20

**Authors:** Jen C. Wang, Hemant Sindhu, Chi Chen, Ajay Kundra, Muhammad I. Kafeel, Ching Wong, Stephen Lichter

**Affiliations:** Division of Hematology/Oncology, Brookdale University Hospital Medical Center, Brooklyn, New York, United States of America; Northwestern University Feinberg School of Medicine, UNITED STATES

## Abstract

Myelofibrosis (MF), including primary myelofibrosis, post-essential thrombocythemia MF, and post-polycythemia vera MF, has been reported to be associated with autoimmune phenomena. IMiDs have been reported to be effective in some patients with MF, presumably for their immune-modulator effects. We therefore sought to elucidate the immune derangements in patients with MF. We found no differences in T regulatory cells (Treg) and T helper 17 (Th17) cells in MF patients and normal healthy controls. However, we found significantly elevated soluble interleukin 2 alpha (sIL2Rα) in MF patients compared to those with other myeloproliferative neoplasm diseases and normal healthy controls. Our studies with MF patients further revealed that Treg cells were the predominant cells producing sIL2Rα. sIL2Rα and IL2 complex induced the formation of Treg cells but not the formation of Th1 or Th17 cells. sIL2Rα induced CD8^+^ T cell proliferation in the presence of Treg cells. Monocytes or neutrophils had no effect on the production of sIL2Rα by Treg cells. Furthermore, we found plasma sIL2Rα levels were correlated to the auto-immune serology in MPN patients and ruxolitinib significantly inhibits the sIL2Rα production by the Treg cells in MF patients which may explain the effects of ruxolitinib on the relief of constitutional symptoms. All these findings suggest that sIL2Rα likely plays a significant role in autoimmune phenomena seen in patients with MF. Further studies of immune derangement may elucidate the mechanism of IMiD, and exploration of immune modulators may prove to be important for treating myelofibrosis.

## Introduction

Myelofibrosis (MF), including primary myelofibrosis (PMF), postessential thrombocythemia post ET MF and postpolycythemia vera PV MF, is characterized by a leukoerythroblastic blood, hepatosplenomegaly, and bone marrow fibrosis. In the early 1980s, studies of immune dysfunction in MF patients showed the presence of circulating immune complex [1,2] and various autoimmune phenomena such as a positive antinuclear antibody test [3], positive Coombs test [4], and presence of lupus-like circulating anticoagulants [5]. Recently, clinical benefits have been reported in patients receiving therapy with thalidomide or lenalidomide [6,7,8]; benefits are presumably derived from immune-modulating effects of these agents, but the exact mechanism remains unclear. Therefore, we proposed to probe further into immune dysfunction in MF.

In cancer patients, increased numbers of T-regulatory (Treg) cells have been observed in peripheral blood, the tumor microenvironment, and in tumor-draining lymph nodes. Studied *in vitro*, these Treg cells display a suppressive immune capacity [9]. Many reports have demonstrated increased numbers of Treg cells in solid tumors, including melanoma [10], gastric carcinoma [11,12], ovarian cancer [13], squamous cell carcinoma of the head and neck [14], and hepatocelluar carcinoma [15]. Also, abundant T immunosuppressive cells have been found in hematologic malignancies such as in Hodgkin’s lymphoma [16,17], chronic lymphocytic leukemia (CLL) [18,19], non-Hodgkin’s lymphoma [20], acute myeloblastic leukemia [21], multiple myeloma [22], and myelodysplastic syndrome [23]. Essentially, Treg cells modulate immune function as follows: Treg cells modulate immune response to infectious pathogens [24], and Treg cells suppress the autoreactive T cell response in the adaptive immune system by maintaining immunological self-tolerance [25]. This suppression is important in preventing autoimmunity in allogenic bone marrow transplantation. Augmented Treg responses can compromise protective immunity against tumors. Thus, Treg cells play an important role in controlling autoimmunity as exemplified by the mutations in FOXP3 resulting in an autoimmune syndrome termed immune dysregulation, polyendocrinopathy, enteropathy, X-linked (IPEX) syndrome [26].

T helper 17 (Th17) cells were first recognized in 1995 as a new set of T helper cells [27]. Since then, Th17 cells have been shown to play a crucial role in the development of inflammatory diseases and autoimmune diseases. In studies of mice that genetically specifically lacked IL-23 or IL-12, the loss of IL-23 made the animals highly resistant to the development of autoimmunity and inflammation, whereas the loss of IL-12 did not [28,29], suggesting that Th17 cells are more important than Th1 cells in the development of autoimmunity. IL-17 has been reported to be increased in cancer including gastric [30], ovarian [31], and head and neck [32], as well as in hematologic malignancies such as acute leukemia [33].

IL-2 exerts its effect through binding to its receptor on cell surfaces. IL-2 receptor (IL-2R) consists of three chains that include the alpha (CD25), beta (CD122), and gamma (CD132) chains [34]. Both beta and gamma chains are constitutively expressed on lymphocytes and have long cytoplasmic domains that activate the cytoplasmic proteins of the JAK-STAT pathway following the binding of IL-2 to the trimeric receptor. The alpha chain is inducible, and high levels of CD25 expression on CD4 T cells are seen after IL-2 activation through the T cell receptor. The main functions of CD25 are to bind IL-2 and to promote optimal IL-2 signaling through the high affinity IL-2R upon its association with the beta and gamma chains. The truncated, soluble form of IL-2R (sIL2Rα) that is generated exclusively by the proteolytic cleavage of the alpha chain was found to be elevated and to play a role in modulating immune response in patients with a variety of autoimmune diseases such as rheumatoid arthritis, multiple sclerosis, systemic lupus erythematosus, scleroderma [35], and various types of cancer.

Therefore, Treg cells, Th17 cells, and sIL2α are important in the modulating immune response, especially in autoimmune diseases and cancer. We studied these cells and cytokines in patients with MF to explore the immune defects present in this disease.

## Materials and Methods

### Patients

We studied 61 patients (27 males and 34 females) with a diagnosis of MF (n = 41), including PMF (n = 33), post-ET MF (n = 5), and post-PV MF (n = 3), and other myeloproliferative disease (MPD) patients, including PV (n = 15) and ET (n = 5). 18 normal volunteers were used as controls. Diagnosis was made according to the World Health Organization classification. The study was approved by the Brookdale Hospital Medical Center Institutional Review Board. All patients gave written informed consent according to the Declaration of Helsinki. The clinical characteristics are shown in Table 1. MF and other MPD patients were older than the volunteer controls. 13 MF patients were on hydroxyurea; 28 patients were not. Patients taking IMiD medications, such as pomalidomide, lenalidomide, or thalidomide, were excluded from the study.

**Table 1:** Patient Characteristics.

Diagnosis	Number of Samples	Age	Gender	Treatment	JAK2 (V617F)(+)
PMF	33	68 (39–85)^a^	M (14) F(19)^b^	Hydrea (4)^c^	14^d^
ET- MF	5	54 (39–60)	M(2) F(3)	Hydrea (3)	4
PV-MF	3	71 (61–89)	M(1) F(2)	Hydrea (1)	3
ET	5	73 (64–80)	M(0) F(5)	Hydrea (3)	2
PV	15	63 (40–93)	M(10) F(5)	Hydrea (1)	13
Control	15	45 (31–70)	M(7) F(8)	None	None

^a^ mean (range of age),

^b^ M, Male; F, Female; Paracentesis, numbers of patients or controls,

^c^ Hydrea, Hydroxyurea; paracentesis, numbers patients,

^d^ numbers of patients

### Auto-immune serology studies

Serum samples obtained from MPN patients were sent for auto-immune serology studies to the commercial laboratory of either Quest Diagnostics or BioReference. The following auto-immune serology by standard techniques were studied: anti-nuclear (ANA) by indirect immunofluorescence on HEp-2 cells; anti-mitochondrial (AMA) by indirect immunofluorescence assay; anti-DNA antibodies by indirect immunofluorescence on Crithidia luciliae cells; anti-extractable nuclear antigen (ENA, in particular, SS-A, SS-B, Sm, RNP, Scl-70) and anticardiolipin IgG and IgM (ACA) by ELISA; anti-thyroglobulin (anti-Tg) and anti-thyroid peroxidase (anti-TPO) by fluoro-immunoenzymatic assay; antibodies lupus-like anticoagulant (LLAC) by dilute Russell viper venom test (DRVVT) and a PTT or LA-sensitive PTT (PTT-LA), one that uses low levels of phospholipid reagents. Direct Coombs test for the detection of IgG and complement bound to RBCs was performed using the tube technique employing the standard method with polyspecific anti-human globulin (anti-IgG+C) and monospecific anti-IgG and anti-C3 antisera.

### Isolation of CD4^+^, CD14^+^, CD20^+^, CD4^+^CD25^+^, CD4^+^CD25^-^ cells, and neutrophils

Mononuclear cells were isolated from peripheral blood through Ficoll-hypaque density gradient column and selected for CD34^+^ cells by using CD34^+^ microbeads (Miltenyi Biotec, Auburn, CA). The CD34^-^ cells obtained from the columns were the source of CD4^+^CD25^+^ and CD4^+^CD25^-^ cells. Cells were isolated using the Regulatory T Cell Isolation Kit (Miltenyi Biotec) according to the manufacturer’s instructions. The CD14^+^ and CD20^+^ cells were also selected from CD34^-^ cells by using the CD14^+^, or CD20^+^ cell Isolation Kit (Miltenyi Biotec). Purity of the isolated cell fractions was checked by flow cytometry. Neutrophils were isolated by lysing the bottom red blood cells after Ficoll-Paque density gradient column separation.

### Flow cytometric analysis and quantification of Treg, Th17 cells

Mononuclear cells were separated from peripheral blood. 10^6^ mononuclear cells were stained for flow cytometry analysis by using the Treg Detection Kit (Miltenyi Biotec, Auburn, CA). For the Th17 cell assay, cells were cultured in Iscove's modified Dulbecco's media (IMDM) with fetal bovine serum and stimulated with PMA (20 ng/ml) for 4h in the presence of ionomycin (1 μg/ml) and monensin (1 μM) before harvesting. The cells were fixed, permeabilized, and immunostained with Treg Detection Kit. Flow cytometric analysis of all specimens were carried out by using cytometric instrument either FACScan equipped with a second 635 laser beam or FASCalibur (BD Bioscience, San Jose, California) in the core facility at Memorial Sloan-Kettering Cancer Center, New York, NY. CALIBRITE 3 and APC beads were used to control the flow cytometric instruments and color compensation was carried out by using each individual fluorescein-conjugated antibody and matched isotype control. 7-aminoactinomycin D (7AAD, 6 ug/ml) was used to exclude dead cells for eliminating nonspecific antibody binding. In general, 50,000 events per specimen was acquired and the acquired flow were further analyzed using FlowJo software (Tree Star.; Ashland, OR). The numbers of Treg cells was calculated as the percentage of CD4^+^ CD25^+^ FoxP3^+^ T cells (Treg) from the number of gated CD4^+^ cells. The Th17 cells were assayed using the Human Th17 Flow Kit (BioLegend, San Diego, CA) for CD3^+^CD4^+^IL-17^+^ cell quantification by flow cytometry calculated as a percentage of gated CD4^+^ cells.

### ELISA of sIL2Rα

Plasma from blood and the medium from the cell culture for CD4^+^, CD8^+^, CD14^+^, and CD4^+^CD25^+^ cells was collected and stored at -80°C for subsequent use. Cells were cultured in 200-l medium and stimulated with either PHA (5 μg/ml) or Dynabeads Human T Cell Activator CD3CD28 (Invitrogen, Grand Island, NY) for 2 days at 37°C in 5% CO_2_. The supernatants were harvested, stored at -80°C, and later analyzed by CD25 ELISA. The collected cell culture medium and peripheral plasma (100 μl each) were applied to BD OptEIA set ELISA kit for sIL2Rα. The levels of sILRα were calculated against a standard curve using recombinant human sIL2Rα.

### Effects of sIL2Rα on Th1, Th17, and Treg cells

CD4^+^ cells were cultured 5–7 days in Iscove's modified Dulbecco's media containing IL-2 (100 μg/ml) and Dynabeads Human T Cell Activator CD3CD28 (Invitrogen) with or without sIL2Rα (Pepro Tech, Rocky Hill, NJ). The cells were then stimulated with PMA (20 ng/ml) for 4h in the presence of ionomycin (1 μg/ml) and monensin (1 μM) before being harvested. IFN-γ secreting cells were immunostained with IFN-γ catch and detection reagents for flow cytometry (Miltenyi Biotec) according to the manufacturer’s protocol. The cultured and PMA-stimulated CD4 cells were also fixed, permeabilized, and immunostained with the Treg Detection Kit (Miltenyi Biotec) for CD4^+^CD25^+^FoxP3^+^ cell quantification and the with Human Th17 Flow Kit (BioLegend, San Diego, CA) for CD3^+^CD4^+^IL-17^+^ cell quantification by flow cytometry.

### XTT cell proliferation assay

CD4^+^CD25^+^ cells were cultured with CD4^+^CD25^-^ cells for 7 days in DMEM containing 10% heat-inactivated FCS, 2 mM L-glutamine, 2 x 10^5^ Dynabeads Human T Cell Activator CD3CD28 (Invitrogen) with or without sIL2Rα (500 ng/ml; Pepro Tech). At the end of culture, XTT labeling reagent was added and incubated 4 h at 37°C, 6.5% CO_2_ before measuring the spectrophotometric absorbance at 450 nm. Proliferative responses were expressed as the values of CD4^+^CD25^-^ T cells cultured alone in the absence of CD4^+^CD25^+^ T cells and were used as 100% nonsuppression control. The percentage inhibition was calculated as the relative difference in proliferative response between CD4^+^CD25^-^ co-cultured with CD4^+^CD25^+^ T cells and CD4^+^CD25^-^ T cell cultures alone.

### CFSE cell proliferation assay

Viable 10^6^ CD4^+^ or CD8^+^ cells were labeled with carboxyfluoresceinsuccinimidyl ester (CFSE, Invitrogen, Carlsbad, CA) at a concentration of 1 μM/10^6^ cells for 10 min at 37°C. Labeling was terminated by adding an equal volume of 100% FBS. After four washes in 10% FBS in complete media, cells were cultured alone or with sIL2Rα and unlabeled Treg cells at a 1:1 (T responder:Treg) ratio and stimulated with 50 μl (7.8 μl of beads suspended in 1 ml PBS and 4 ml complete media) of anti-CD3CD28-coated microbeads for 5 days at 37°C in 5% CO_2_ incubator. CD4^+^ and CD8^+^ cells in medium alone served as controls. On day 5, cells were washed twice, and cell division was analyzed in all culture conditions. Cells undergoing division were identified by the percentage of CFSE^dim^ cells.

### Effects of ruxolitinib on sIL2Rα production by T cells

CD4+CD25+ cells were isolated from peripheral mononuclear cells using a T regulatory Cell Isolation Kit (Miltenyi Biotec Inc, Auburn, CA) and cultured for 7 days at 37°C, 5% CO2, in IMDM containing 3% FBS with CD3/CD28 microbeads (Invitrogen Corp; Carlsbad, CA). The cells were treated with Ruxolitinib (3 uM; SelleckChem, Houston, TX) or equivalent volume of its vehicle, DMSO every other days until they were harvested To harvest, the culture medium was spun and supernatants collected and stored at -80°C, before use. Human sIL2Rα ELISA was performed using OptEIA Set for human IL-2sRα ELISA from BD Biosciences (San Diego, CA) as described.

### Effects of monocytes and neutrophils on the production of sIL2Rα by Treg cells

Treg cells were cultured with various concentrations of CD14^+^ or neutrophils in 200 μl medium and stimulated with Dynabeads Human T Cell Activator CD3CD28 (Invitrogen) for 2 days at 37°C in 5% CO_2_. The supernatants were harvested, stored at -80°C and later analyzed by CD25 ELISA.

### Statistical analysis

Statistical analyses were performed with paired or unpaired Student’s *t*-test and one-way ANOVA for comparison of groups. Results are given as mean with standard error and 95% confidence intervals (CIs). Statistical analysis was with Mann–Whitney U-test or Willcoxan signed-rank, for nonparametric data (GraphPad Prism version 5.0, San Diego, CA). Data are expressed as median, (interquartile range). *p*<0.05 was considered significant.

## Results

### Treg cells in patients with MF compared with patients with other MPN diseases and controls

**Fig 1:**
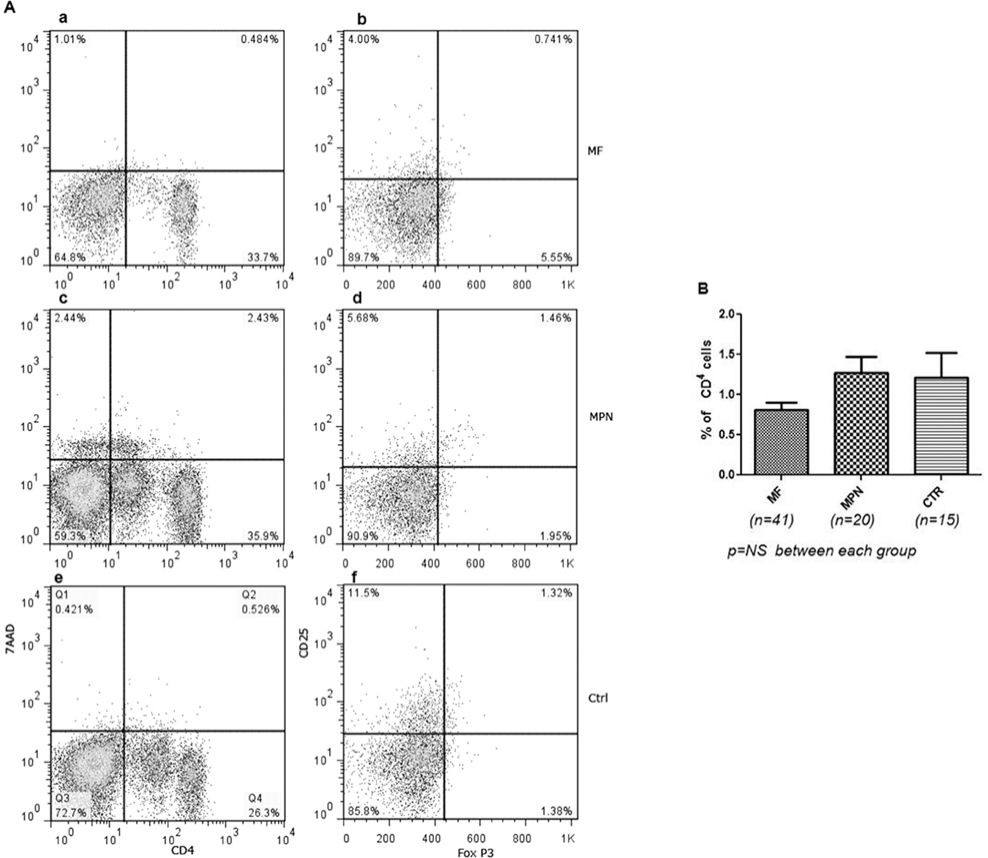
Treg cells in patients with MF and other MPNs. 41 patients with MF including PMF (n = 33), post-ET MF (n = 5), and post-PV MF (n = 3), and other MPN patients including PV (n = 15) and ET (n = 5) were studied. 15 normal volunteers were used as controls. Mononuclear cells from peripheral blood obtained from patients were analyzed by flow cytometry with the T regulatory Detection Kit. (A) Representatives of flow cytometric analysis of Treg cells in peripheral MNC. The viable CD4+ cells (lower-right quadrant) in inserts a, c, and e were further analyzed for CD25+ FoxP3+ cells (upper-right quadrant in inserts b, d, and f). The number of Treg cells was calculated as the percentage of CD4^+^CD25^+^FoxP3^+^ T cells (Treg) from the number of gated CD4^+^ cells. (B) Comparison of Treg cells in MF patients with other MPD patients and controls. No significant difference was found between the groups. MF = myelofibrosis, MPN = myeloproliferative neoplasm, CTR = control.

**Fig 2:**
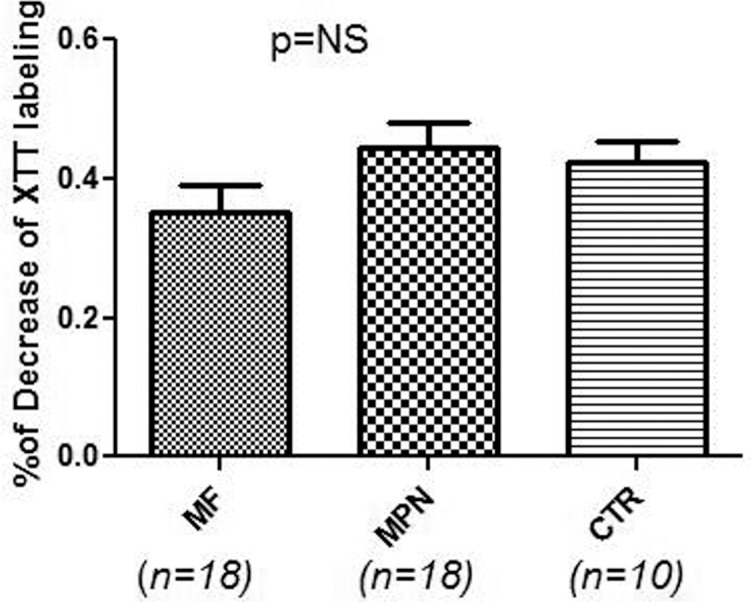
Function of regulatory T cell in MF patients. Treg function was measured as the percentage of suppression of cell proliferation of CD4^+^CD25^-^ by CD4^+^CD25^+^ cells using an XTT-based colorimetric assay. CD4^+^CD25^-^ cells were cultured with CD4^+^CD25^+^ cells, Dynabeads Human T Cell Activator CD3CD28 (Invitrogen) were added for 7 days, XTT-labeled reagent was added and incubated for 4 h at 37°C, 6.5% CO_2_, and spectrophotometric absorbance was then measured at 450 nm. The values of suppression are expressed as percentage of the values of suppression of proliferation response using CD4^+^CD25^-^ T cells cultured alone in the absence of CD4^+^CD25^+^ T cells and were used as 100% of nonsuppression control. MF = myelofibrosis, MPN = myeloproliferative neoplasm, CTR = control.

We started to study Treg cells in MF and MPD patients in 2009. We have sequentially studied 41 patients with MF including 33 patients with PMF, 5 patients with post-ET MF, and 3 patients with post-PV MF; 20 patients with other MPN diseases including 15 patients with PV and 5 patients with ET (there are no apparent differences between ET and PV, so they were grouped together); 18 normal volunteers were used as controls. Age and other characteristics including treatment with hydroxyurea and Jak2 V617F mutation are presented in Table 1. Treg cells are presented as a percentage of CD4^+^CD25^+^Foxp3^+^ of CD4^+^ (Fig. 1). There were no significant differences in the number of Treg cells between the different groups. We also compared the patients who were on hydroxyurea at the time of study with those patients who were not on the drug (data not shown). There were no differences between the two groups. Treg cells were evaluated in patients with MF relative to the difference between negative and positive Jak2 V617F; no differences were found (data not shown). We further studied the Treg function in MF patients compared with other MPN patients and normal controls: Treg function was measured as a percentage of decrease of XTT with T response cells (CD4^+^CD25^-^) cultured with Treg cells (CD4^+^CD25^+^) and as a percentage of cultured medium alone. No significant difference was found (Fig. 2).

### Th17 cells in MF compared with other MPNs and controls

Th17cells were counted as the percentage of CD3^+^CD4^+^IL-17^+^ cells from CD4^+^ cells after stimulation. 15 MF patients including 2 patients with post-ET MF, 3 patients with post-PV MF, 10 patients with PMF; 7 patients with other MPN diseases including ET ([4] and PV [3]; and 10 normal volunteer controls were studied. No significant difference was found among these groups, (data not shown).

### Plasma sIL2Rα levels

**Fig 3:**
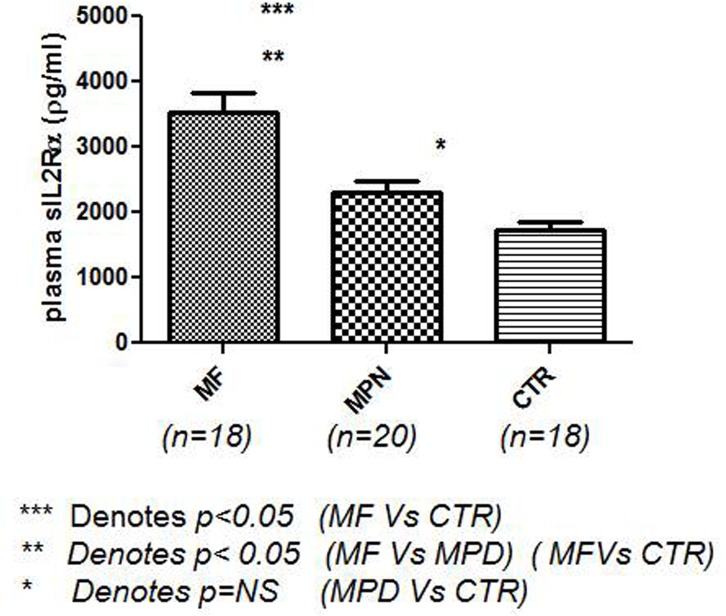
Plasma sIL-2Rα levels in Patients with MF and others. Levels of sIL2Rα in peripheral plasma were quantified using BD OptEIA ELISA set for human sIL-2Rα. MF patients had a significantly elevated sIL-2Rα compared with other MPN patients and controls. Other MPN patients were not significantly different from controls. MF = myelofibrosis, MPN = myeloproliferative neoplasm, CTR = control. ***P<0.0001, **P< 0.0001, NS = no significance.

We previously found plasma sIL2Rα levels were significantly elevated in patients with MF compared with MPN patients and controls [36]. We repeated the study and found that MF patients had significantly elevated plasma sIL2Rα levels compared with other MPN patients and controls (Fig. 3).

### Treg cells are responsible for elevated sIL2Rα in MF patients

**Fig 4:**
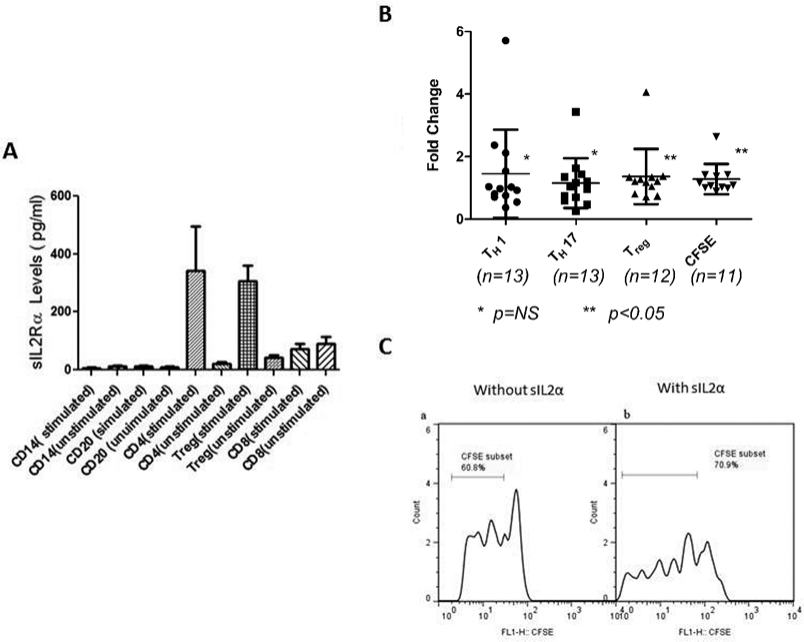
sIL2Rα and T cells. (A) Treg cells release sIL2Rα in MF patients. Immune cell types were purified from peripheral blood mononuclear cells from MF patients by using magnetic antibody cell sorting beads for CD4^+^ T cells, CD8^+^ T cells, Treg cells, CD14^+^ monocytes, and CD20^+^ B cells. Cells were then cultured for 2 days in a 96-well plate and were stimulated with either anti-CD3CD28 beads or PHA; supernatants were then assayed for sIL2Rα. Treg cells were the predominant cells producing sIL2Rα. (B) Effects of sIL2Rα on the proliferation and differentiation of CD4^+^T cells in MF patients. CD4^+^ cells were cultured with IL-2 (10 ng/ml) with and without sIL2Rα (100 ng/ml) 5–7 days and then assayed for Th1, Th17, and Treg cells by flow cytometry. The effects were calculated as the fold-change with stimulated over unstimulated sIL2α. Data were presented as median ± interquartile range. As shown in Fig. 4b, sIL2Rα stimulated formation of Treg cells and stimulated the proliferation of CD4^+^ T cell proliferation as measured by CFSE staining but had no effect on differentiation to Th1 and Th17 cells. (C) Representative screens of flow cytometric analysis of cell proliferation with CFSE using peripheral CD4+ cells from patients with PMF with (b) or without (a) sIL-2Rα.

Isolated cells were stimulated either with T cell activator CD3CD28 microbeads or PHA and cultured 1–3 days. Supernatant was then analyzed by ELISA. CD4^+^ and Treg cells produced significantly higher amounts of sIL2Rα compared to other cells (Fig. 4a). Therefore, Treg cells are predominantly responsible for elevated sIL2α in MF patients.

### Effects of sIL2Rα on the proliferation and differentiation of CD4^+^ T cells

CD4^+^ cells were cultured with IL-2 with and without sIL2Rα for five-to seven days and then assayed by flow cytometry for Th1, Th17, and Treg cells. The effects were calculated as the fold-change of the sIL2Rα-stimulated over un-stimulated cells. sIL2Rα stimulated formation of Treg cells (median, 1.21, interquartile range (0.86–1.34), p = 0.02) and stimulated the proliferation of CD4^+^ T cells (median,1.09, interquartile range (1.009–1.41), p = 0.03), but had no effect on differentiating Th1 and Th17 cells (Fig. 4b, c).

### Effects of sIL2Rα on the proliferation of CD8^+^ T cells in the presence of Treg cells

**Fig 5:**
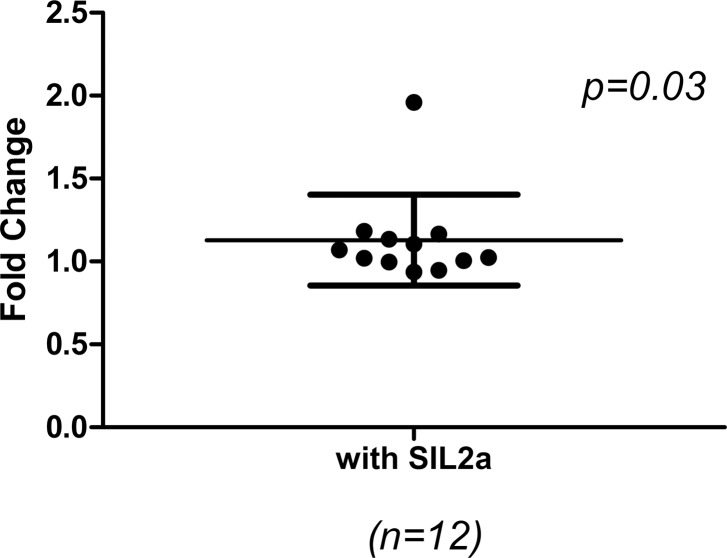
Effects of sIL2Rα on the proliferation of CD8^+^ T cells in the presence of Treg cells. To investigate the effects of sIL2Rα on proliferation when CD8^+^ T cells were co-cultured with Treg cells, CD8^+^ T cells (10^5^/ml) were co-cultured with CD4^+^CD25^+^ (10^5^/ml) then stimulated with T cell activator CD3CD28 microbeads and sIL2Rα (100 ng/ml) 5–7 days. Percentage of CFSE^dim^ cells were counted as the proliferation of CD8^+^T cells. The results were calculated as the fold-change of the sIL2α-stimulated over unstimulated cells. sIL2Rα induced CD8^+^T cell proliferation (median, (interquartile range) of 1.04, (0.99–1.15), p = 0.02) when they were co-cultured with Treg cells.

To investigate the effects of sIL2Rα on proliferation when CD8^+^ T cells were co-cultured with Treg cells. CD8^+^T cells were co-cultured with Treg cells and then stimulated with T cell activator CD3CD28 microbeads and sIL2Rα for 5–7 days. Percentage of CFSE^dim^ cells was determined as the proliferation of CD8^+^T cell proliferation. The results were calculated as the fold-change of the sIL2α-stimulated over un-stimulated cells. sIL2Rα induced CD8^+^ T cell proliferation (Median,1.04, interquartile range (0.99–1.15), p = 0.02) when co-cultured with Treg cells (Fig. 5).

### Correlation of sIL2Rα and autoimmune serology

**Fig 6:**
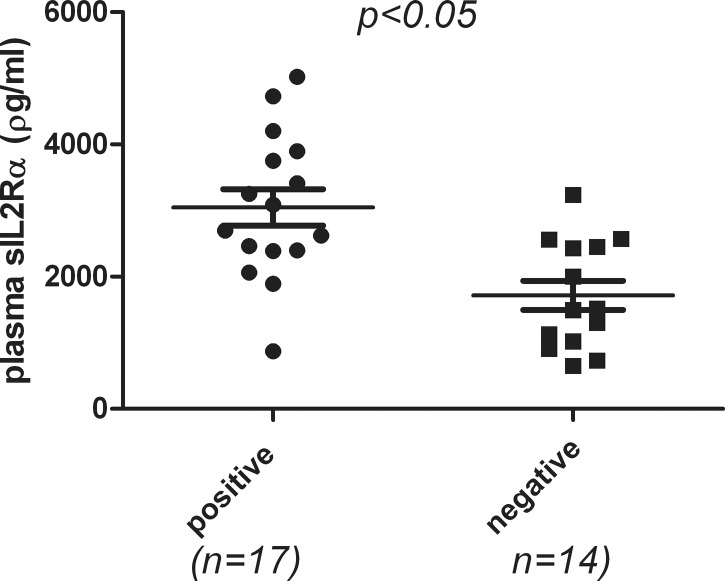
Correlation of auto-immune serology and sIL2Rα levels in patients with MPN diseases. Auto-immune serology panels were performed as outlined in the materials and methods. 31 patients including PMF (9) ET-MF(1) PV-MF(4), ET(8), PV(9) were studied. Patients with at least one positive serology have significantly elevated sIL2Rα levels compared to negative patients (p<0.05)

31 patients including PMF (9) ET-MF(1) PV-MF(4), ET(8), PV(9) were studied. Nine MF patients had positive serology including two with ANA(+), four with positive anti-cardiolipin antibody, two with positive lupus-anticoagulant.and one with positive to thyroglobulin. Four ET patients had positive serology including two with positive ANA, one with positive rheumatic factor, and one with positive cardiolipin antibody. Four PV patients with positive serology including one with positive ANA, one positive anti-thyroglobulin, and two with positive anti-cardiolipin antibody. As shown in Fig. 6, 17 patients who were positive for any one of the auto-immune serology were compared with 14 patients with negative serology. Patients with positive serology have significantly elevated sIL2Rα levels compared to negative patients (p<0.05).

### Effects of ruxolitinib on the production of sIL2Rα by Treg cells

**Fig 7:**
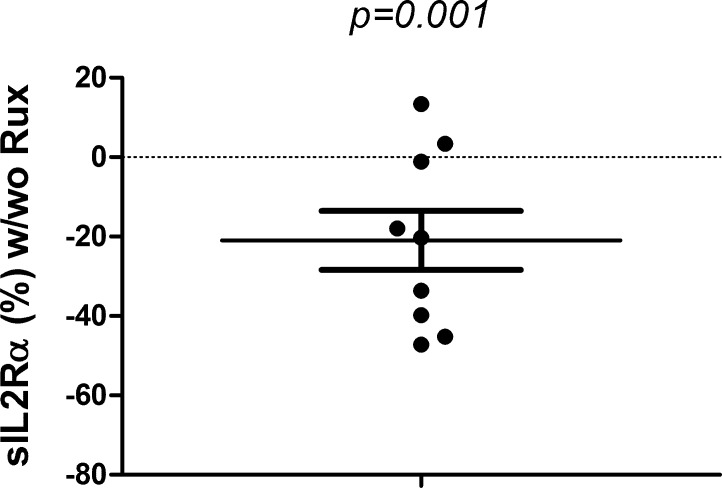
Effects of ruxolitinib on the production of sIL2Rα by Treg cells. CD4+CD25+ cells were isolated from peripheral mononuclear cells using T regulatory Cell Isolation Kit and cultured for 7 days at 37°C, 5% CO_2_, in IMDM containing 3% FBS with CD3/CD28 microbeads The cells were treated with Ruxolitinib (3 uM; SelleckChem, Houston, TX) or equivalent volume of its vehicle, DMSO every other days till it was harvested. To harvest, the culture medium was spun and supernatants collected and stored at -80°C, before use. Human sIL2α ELISA was performed using OptEIA Set for human IL-2sR α ELISA from BD Biosciences. 9 patients (7 PMF, 1 each for PV-MF, and ET-MF) were studied. Changes of sILR2α were expressed as the percentage of values of of sIL2Rα produced with ruxolitinib by the values without ruxolitinib. Ruxolitinib significantly inhibits (%) the production of sIL2Rα by the Treg (mean ± SE, 20.94 ± 7.42), (p = 0.001) (n = 9).

9 patients (7 PMF, 1 each for PV-MF, and ET-MF) were studied. Changes of sILR2α were expressed as the percentage of values of sIL2Rα produced with ruxolitinib by the values without ruxolitinib. As shown in Fig. 7, Ruxolitinib significantly inhibits (%) the production of sIL2Rα by the Treg (mean±SE, 20.94±7.42) (p = 0.001) (*n = 9)*.

### Effects of monocytes or neutrophils on the production of sIL2Rα by Treg cells

To elucidate the mechanism of increased production of sIL2Rα in MF patients, Treg cells were co-cultured with monocytes or neutrophils and stimulated with CD3CD28 beads from MF patients. No differences in sIL2Rα levels were detected in Treg cells cultured with or without monocytes or neutrophils (data not shown), suggesting that monocytes and neutrophils have no significantly stimulating effects on Treg cells in producing sIL2Rα.

## Discussion

Through the release of CCL22/CCR4 and PGE_2_ or H-ferritin, tumor cells, including those in hematologic malignancies, cause attraction and expansion of Treg cells to the tumor site and in peripheral blood (PB), thereby increasing Treg cells in the tumor microenvironment and in PB [37]. Because patients with myelofibrosis have been found to have associated autoimmunity, beginning in 2009, we analyzed T cell immunity in myelofibrosis patients and found that Treg numbers were not altered compared to normal volunteer controls [38] (Fig. 1a). Riley et al. subsequently reported the same finding: no changes in Treg cells in MPN disease patients [39]. We also found no difference in Treg cells in MF patients on hydroxyurea versus untreated patients (the same results as reported by Riley et al [39]. We also demonstrated no statistically significant changes of Treg cell function in MF patients compared with controls (Fig. 2).

We repeated the sIL2Rα assay studies and reconfirmed our previous finding that sIL2Rα is significantly elevated in MF patients MF compared with other MPN disease patients and controls (Fig. 3). Clinically, high sIL2Rα serum levels have been found in cases of autoimmune diseases and malignancies and infectious diseases, [40]. Many studies have measured serum sIL-2Rα levels and established its potential value as a prognostic factor in B-cell non-Hodgkin’s lymphoma, especially in aggressive subtypes such as diffuse large B-cell lymphoma [41]. We previously reported that sIL2Rα levels correlate with overall survival of MF patients [36]. Therefore, sIL2Rα may play an important role in autoimmunity and prognosis in MF patients.

We further tried to elucidate which cells are responsible for the production of sIL2Rα. CD4^+^ T cells and Treg cells and not CD14^+^ or CD20^+^ cells were the predominant cells producing sIL2Rα (Fig. 4a). This finding is similar to that in a study involving B cell malignancies where Treg cells were shown to be the predominant cells producing sIL2Rα [42].

We also explored the effects of sIL2Rα on immune response. The sIL2Rα–IL-2 complex promoted T cell differentiation toward Treg cells rather than toward Th1 or Th17 (Fig. 4B and C), similar to findings reported by Yang et al. [43] that IL2Rα-IL-2 complex promoted T cell differentiation toward Treg cells in follicular B cell non-Hodgkin’s lymphomas. The increased proliferation of CD4^+^ T cells by the sIL2Rα-IL-2 complex by the CFSE assay (Fig. 4c) further confirmed that sIL2Rα-IL-2 complex promotes the CD4^+^ formation and proliferation.

We further studied the effects of sIL2Rα on the interaction between Treg and CD8^+^ T cells. sIL-2Rα significantly induced the proliferation of CD8^+^ T cells, in the presence of Treg cells in cultures (Fig. 5). sIL2Rα-IL-2 complex significantly inhibited the proliferation of blood mononuclear cells in B cell malignancies (44) which have different findings from ours. The difference in findings may be that mononuclear cells were used in the Lindqvist et al. study [42]) in contrast to our study where CD8^+^ T cells were the target cells. In addition, IL-2 was not added to the cultures in our study. The of Maier et al. [44] that sIL2Rα alone can induce T cell proliferation and response are similar to ours. We conclude that sIL2Rα attenuates Treg function and induces CD8^+^ T cell proliferation. These results may explain the autoimmune phenomena seen in some patients with myelofibrosis.

Autoimmune phenomena, or serology without clinical evidence of connective tissue disease in myelofibrosis, was described 20 years ago [45]. In a recent more comprehensive study by Barcellini et al [46], anti-erythrocyte antibodies by mitogen-stimulated direct antiglobulin test (MS-DAT) were positive in 45%, anti-platelets in 15% and organ/non organ-specific autoimmune serology in 57% of cases and up to 87% in early myelofibrosis cases, all were without clinically overt disease. We studied 31 patients with MPN disease and found patients with at least one positive autoimmune serology have significantly elevated sIL2Rα than those with negative serology as shown in Fig. 6. All these patients have no clinical overt evidence of auto-immune diseases. These findings plus the in vitro data (Figs. 4 b, c, and 5) suggest that sIL2Rα are possibly related to the autoimmune phenomenon in patients with myelofibrosis. The robustly elevated levels of sIL2Rα observed in MF patients with the lack of overt associated auto-immune diseases maybe due to other counter-balance mechanisms. We had found an increased Myeloid Derived Suppressor Cells (MDSC) population in patients with MF [47]. Further studies will be necessary to solve this complex issues.

Ruxolitinib significantly improves constitutional symptoms [48,49] and has been approved for the treatment of MF. Constitutional symptoms are related to the inflammatory cytokine including sIL2Rα, IL8, and IL15 among others [50]. Fig. 7 shows that ruxolitinib significantly inhibits the sIL2Rα produced by the Treg cells in MF patients, consistent with clinical improvement of constitutional symptomatology with ruxolitinib. A further in vitro or in vivo testing of the inhibitory effects to the other cytokines by ruxolitinib will need to be done to substantiate this mechanism.

We explored the mechanism of increased production of sIL2Rα in patients with MF; monocytes or neutrophils were co-cultured with Treg cells, but no significant stimulating effects were detected (data not shown). Studies including the decreased apoptosis of Treg cells or increased expression of PD1 are ongoing to explore the mechanism of increased production of sIL2Rα in MF patients.

Promising immunotherapies have been developed for cancer: ipilimumab (CTLA-4 antibody) for melanoma [51] and PD-1 and PD-L1 antibody for melanoma, renal cell carcinoma, and lung cancer [52, 53]. In MPN diseases, interferon-α 2a (IFN-α 2a) has been revived and promising results have been observed [54,55] in which IFNα 2a was shown to exert potent effects on Treg cells [39]. The JAK inhibitor, Ruxolitinib, which has been approved for the treatment of myelofibrosis was also found to have potent immune inhibitory effects on dendritic cells [56]. Because no other treatment modalities except bone marrow transplantation have been found to be a possible curative treatment in MF, further exploration of immunotherapy may be likely to open a new era in the treatment of MPN diseases in the future. Further exploration of basic immune pathology in myelofibrosis may shade light on treating this incurable disease.
